# A randomized, double‐blind clinical trial to evaluate the blood pressure lowing effect of low‐sodium salt substitution on middle‐aged and elderly hypertensive patients with different plasma renin concentrations

**DOI:** 10.1111/jch.14396

**Published:** 2021-12-29

**Authors:** Li Che, Wei Song, Ying Zhang, Yan Lu, Yunpeng Cheng, Yinong Jiang

**Affiliations:** ^1^ Department of Cardiology First Affiliated Hospital of Dalian Medical University Dalian Liaoning China

**Keywords:** aldosterone, home blood pressure, office blood pressure, renin, salt substitution

## Abstract

This study aimed to evaluate the blood pressure (BP) lowing effect of low‐sodium (LS) salt substitution and how the effect influenced by plasma renin concentration (PRC) on middle‐aged and elderly hypertensive patients. Three hundred fifty‐two hypertensives were randomized at a 1:1 ratio into a LS group and a normal salt (NS) group. We compared intergroup changes observed in office blood pressure measurement (OBPM) and home blood pressure measurement (HBPM). Then, all patients in LS group were divided into tertiles according to baseline PRC, aldosterone concentration, and aldosterone/renin ratio (ARR), and changes in OBPM and HBPM were compared across the three tertile subgroups. Follow‐up surveys were completed by 322 patients. The intergroup net reduction in systolic OBPM, systolic HBPM, and diastolic HBPM was −6.6, −4.6, and −2.3 mmHg, respectively (all *P* < .05), and −1.8 mmHg in diastolic OBPM (*P* = .068). There was a more significant reduction in OBPM and HBPM among the low baseline PRC subgroup than among the high PRC subgroup. There were no significant differences in the changes in OBPM and HBPM between the three subgroups when grouped according to baseline aldosterone concentration. The reduction in OBPM and HBPM in the high tertile of ARR was larger than that in the low tertile subgroup. LS salt substitution is effective in reducing systolic OBPM, systolic HBPM, and diastolic HBPM in middle‐aged and elderly hypertensive patients. LS salt substitution may offer a non‐pharmaceutical therapy for hypertensive patients. Baseline PRC may be a marker to predict BP response after salt restriction.

## 1 INTRODUCTION

High blood pressure (BP) is a major preventable risk factor for heart disease, kidney disease, and cerebral hemorrhage and infarction. Although highly prevalent, the treatment rate (36.9%) and control rate (13.8%) for hypertension are extremely low worldwide.^1^ However, high sodium intake is a modifiable risk factor proven to be positively associated with BP, unlike potassium. A study has demonstrated that each one gram increase in 24‐hour sodium excretion can increase BP by 4.58/2.25 mmHg, while aleach one gram increase in urinary potassium can decrease systolic blood pressure (SBP) by 3.72 mmHg.^2^ Other studies report that reducing sodium intake lowers BP,^3^ while potassium depletion leads to an increase in BP.^4^ At present, global salt intake is approximately 10 g per day—and 12.5 g per day in China^5^—which exceeds the World Health Organization (WHO) 5 g/day recommendation.^6^ Only a few countries meet the daily potassium consumption recommendation proposed by the WHO (upwards of 3510 mg/day).^7^ Recent guidelines emphasize lifestyle modification, especially salt reduction and increasing consumption of potassium‐rich foods, as one of the first choices of antihypertensive therapy.^8^ Several studies have proved salt substitution low in sodium, enriched in potassium and sometimes combined with minerals just like calcium and magnesium may reduce both SBP and diastolic blood pressure (DBP).^9^, ^10^, ^11^

Research has proved that compared to office blood pressure measurement (OBPM), home blood pressure measurement (HBPM) can be measured more accurately and it is a stronger predictor of cardiovascular outcome,^12^ and should be used in the diagnosis and evaluation of hypertension.^8^ While numerous studies have reported the positive effect of low‐sodium (LS) salt substitution on BP, most of these studies were performed using OBPM or HBPM alone, with only a few conducted by monitoring OBPM and HBPM simultaneously.

A previous study demonstrated sodium‐sensitivity of BP was determined by renin^13^; therefore, we evaluated BP reduction with different levels of renin respond to the LS salt substitution intervention.

This study evaluates the effect of low‐sodium salt substitution on OBPM and HBPM in middle‐aged and elderly hypertensive patients, and how the effect influenced by plasma renin concentration (PRC).

## 2 MATERIALS AND METHODS

### 2.1 Participants

We enrolled hypertensive outpatients from two community service centers in Dalian, Liaoning, North China from August, 2019 who met the inclusion and exclusion criteria. Hypertension was defined as having an office SBP of ≥ 140 mmHg and/or a DBP of ≥ 90 mmHg or taking antihypertensive agents.^8^ The inclusion criteria were: (1) Primary hypertensive patients with office SBP from 140 to 180 mmHg and/or DBP from 90 to 110 mmHg measured by trained doctors; (2) Age: 50‐70 years; and (3) Eating at least two home‐cooked meals per day. The exclusion criteria were (1) Secondary hypertension: renal artery stenosis, glomerulonephritis, primary aldosteronism, Cushing's syndrome, sleep apnea syndrome, and pheochromocytoma; (2) Renal insufficiency (eGFR < 60 mL/min/1.73m^2^), acute myocardial infarction, severe heart failure (EF < 50%), or hepatic disease (AST or/and ALT three‐fold limitation); (3) Long‐term bedridden patient; (4) Using diuretics medication; (5) A baseline serum potassium concentration of ≥ 5.0 mmol/L.

### 2.2 Study design

This is a 12‐month prospective, multicenter, randomized, double‐blind study. Using a computerized randomization program, the study participants were randomly assigned to one of two groups: the normal salt (NS) group or the LS group, in a 1:1 ratio. The NS group consumed 100% sodium chloride, while the substitution consumed by the LS group was 43% sodium chloride, 32% potassium chloride, and 25% other ingredients.

The participants were asked to replace the salt they used for cooking with the salt provided by the study. The questionnaire was achieved to assess the ordinary salt intake of every family, which would guide the quantity of salt distributed. Every participant also got a 5‐g spoon to help them control the amount of salt that was informed used during cooking. Telephone follow‐up was conducted every 2 weeks to instruct all the participants to reduce salt during cooking and to assess the use of the salt distributed. The recruiting and assigning work was completed by an assistant who was not in charge of the follow‐up, and neither the investigators nor the participants were informed of the assignment until the end of the follow‐up period.

### 2.3 Follow‐up

**FIGURE 1:**
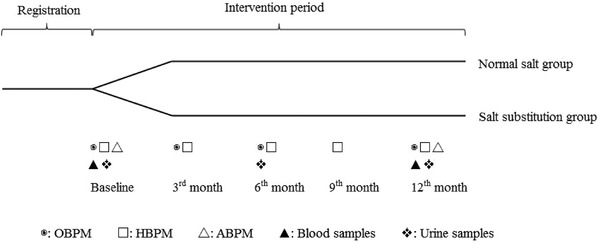
Flowchart of the study

Figure 1 presents the study flow chart. At baseline, all patients underwent a personal interview by trained investigators on demographic characteristics such as age, sex, height, weight, smoking history, alcohol history, and medication history. Body mass index (BMI) was calculated as weight (kg) divided by the square of height (m^2^). The follow‐up survey was scheduled for the 3^rd^, 6^th^, and 12^th^ months. Only the HBPM was taken in the 9^th^ month because of the coronavirus epidemic of 2019.

### 2.4 Blood pressure measurement

The OBPM was performed at the start of the study, and in the 3^rd^, 6^th^, and 12^th^ months. The procedure involved the participants sitting and relaxing for 5 minutes before the measurement was taken using a validated electronic upper‐arm cuff device (Contec08D, Qinhuangdao, China). The BP in the right arm was measured thrice, with the patient in a seated position, at one‐minute interval, and the average of the last two measurements was used as the OBPM.

All patients received free electronic upper‐arm cuff devices (Contec08D, Qinhuangdao, China) to measure their BP at home. The HBPM was taught by specialists to the participants and was performed at the start of the study, and in the 3^rd^, 6^th^, 9^th^, and 12^th^ months. Readings were taken in the morning and evening after participants rested for five minutes in a quiet environment on three consecutive appointed days. Two readings were taken with a 1‐minute interval between each reading, and the average of all measurements taken during the three days was calculated and used as the HBPM.

### 2.5 Biochemistry

Fasting blood specimens were obtained from each participant at the beginning and at the end of the study period, and fasting spot urine specimens were collected at the beginning, and in the 6^th^ and 12^th^ months using urine collection cups and storing tubes. We measured serum sodium, potassium, blood urea nitrogen (BUN), serum creatinine (Scr), uric acid (UA), PRC, aldosterone concentration, urine sodium and potassium levels using the collected samples at the DMU (Dalian Medical University) clinical laboratory. ARR was calculated as the ratio of aldosterone to renin. We used the INTERSALT method to evaluate the 24‐hour urinary sodium excretion and Tanaka method to evaluate the 24‐hour urinary potassium excretion via spot urinary sodium and potassium.^14^, ^15^, ^16^

### 2.6 Outcomes

The primary outcome was the changes of OBPM and HBPM. The secondary outcome was to compare the BP changes across the three tertile subgroups grouped according to baseline PRC, aldosterone concentration, and aldosterone/renin ratio (ARR).

### 2.7 Statistics

On the basis of a prior reference,^17^ the difference in mean SBP between groups was 4.9 mmHg in that study, sample size estimation was done using the formula

$$N=\left(1+1/\kappa \right){\left(\sigma \frac{z1\;-\;\frac{\alpha }{2}\;+\;z1-\beta }{\mu A-\mu B}\right)}^{2}$$

where α was 0.05, and 1 − β was 0.9. The sample size was 160 for each experimental group. Taking into account the possibility of participant loss to follow‐up and other factors, this number was increased by 10%, bringing the final sample size to 352 patients—176 patients in each group.

Statistical analysis was performed using SPSS 25.0. Per‐protocol (PP) analysis was used. Continuous variables with a normal distribution are expressed as mean ± SD and compared using the *t* test. Abnormal distribution variables are reported as median and interquartile ranges and compared using the Mann–Whitney U test. Categorical variables are presented as frequencies and compared using the Chi‐squared test. The Kruskal–Wallis test was used to compare the changes in BP across the subgroups. A value of *P* < .05 was considered statistically significant.

## 3 RESULTS

### 3.1 Demographic characteristics

A total of 352 patients were enrolled, and 30 (8.5%) of these participants withdrew during the intervention stage, while 160 participants in the NS group and 162 in the LS group completed all visits. The average age was 62.17 ± 4.69 years for the NS group, and 62.96 ± 4.51 years for the LS group. The NS group had 72 (45.0%) male patients, while the LS group had 58 (35.8%). At baseline, there were no significant differences in age, sex, BMI, smoking and alcohol history, systolic and diastolic OBPM, and HBPM and antihypertensive medication use (*P* > .05). We observed no differences in baseline PRC, aldosterone concentration, and ARR between the two groups (*P* > .05) (Table 1).

**TABLE 1:** Demographic characteristics of the participants

Characteristics	NS	LS	
	(n = 160)	(n = 162)	*P*
Age (y)	62.17 ± 4.69	62.96 ± 4.51	.125
Male sex (n, (%))	72 (45.0)	58 (35.8)	.093
BMI (kg/m^2^)	25.67 ± 3.19	26.16 ± 3.23	.174
Smoking history (n, (%))	27 (16.9)	28 (17.3)	.922
Alcohol history (n, (%))	21 (13.1)	20 (12.3)	.834
OBPM SBP (mmHg)	133.22 ± 13.04	135.32 ± 13.74	.161
OBPM DBP (mmHg)	78.00 ± 11.18	77.61 ± 10.11	.744
HBPM SBP (mmHg)	129.44 ± 11.41	130.13 ± 10.76	.579
HBPM DBP (mmHg)	78.24 ± 8.74	78.06 ± 8.46	.849
Antihypertensive medication			
CCB (n, (%))	106 (66.3)	109 (67.3)	.844
ACEI/ARB (n, (%))	58 (36.3)	70 (43.2)	.131
β‐blocker (n, (%))	29 (18.1)	24 (14.8)	.423
CCB + ACEI/ARB (n, (%))	33 (20.6)	42 (25.9)	.261
CCB + β‐blocker (n, (%))	19 (11.9)	18 (11.1)	.830
ACEI/ARB + β‐blocker (n, (%))	9 (5.6)	13 (8.0)	.393
CCB + ACEI/ARB + β‐blocker (n, (%))	5 (3.1)	8 (4.9)	.409
PRC (uIU/mL)	11.4 (4.5, 24.0)	11.3 (4.5, 21.6)	.547
Ald (pg/mL)	103.0 (73.0, 150.8)	96.5 (68.2, 145.0)	.538
ARR	8.7 (4.1, 19.0)	9.6 (4.1, 23.8)	.613

NS, normal salt; LS, low‐sodium; Ald, aldosterone.

### 3.2 Comparison of changes in OBPM and HBPM

**FIGURE 2:**
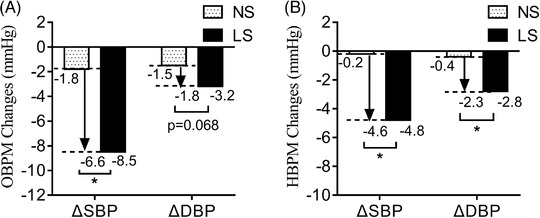
Comparison of OBPM and HBPM changes in PP analysis. PP, Per‐protocol; NS, normal salt; LS, low‐sodium, Δ: change of BP, * *P* < .05

First, we compared the OBPM values and found that the change in SBP among the LS group was significantly greater than that among the NS group [intergroup net reduction −6.6 mmHg ± 1.3 (95% CI −3.9 to −9.2), *P* = .001], whereas the DBP change between the two groups was statistically insignificant [intergroup net reduction −1.8 ± 1.0 mmHg (95% CI −0.2 to −3.7), *P* = .068] (Figure 2A).

Then, we compared the HBPM results and found that the changes in both SBP and DBP among the LS group were larger than those in the NS group: intergroup net reduction −4.6 ± 1.4 mmHg (95% CI −1.8 to −7.4), *P* = .003 for the change in SBP; −2.3 ± 1.0 mmHg (95% CI −0.6 to −4.3), *P* = .021 for DBP change (Figure 2B).

### 3.3 Comparison of changes in OBPM for different subgroups according to baseline PRC, aldosterone concentration, and ARR

Changes in BP were −13.0 ± 1.3/−5.0 ± 1.0 mmHg in the first tertile of the PRC subgroup (PRC: ≤ 5.896 uIU/mL), −7.3 ± 1.6/−3.3 ± 1.0 mmHg in the second tertile (PRC: 5.896–16.650 uIU/mL), and −4.8 ±1.6/0 ± 1.1 mmHg in the third tertile (PRC: > 16.650 uIU/mL). BP fell more among the low PRC group than among the high PRC group (*P* < .001 for change in SBP, and *P* = .001 for change in DBP).

Among the aldosterone subgroups, the changes in BP were −6.3 ± 1.5/−3.3 ± 1.1 mmHg in the first tertile (Ald: ≤ 81.500 pg/mL), −7.8 ± 1.8/−2.0 ± 1.1 mmHg in the second tertile (Ald: 81.500–130.000 pg/mL), and −8.8 ± 1.4/−3.3 ± 0.9 mmHg in the third tertile (Ald > 130.000 pg/mL). There were no significant differences between the three subgroups (*P* = .447 for change in SBP, and *P* = .770 for change in DBP).

**FIGURE 3:**
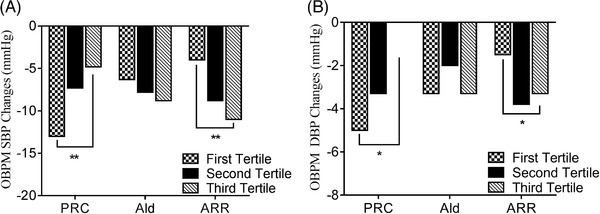
Comparison of OBPM changes in different subgroups according to baseline PRC, Ald, and ARR. Ald, aldosterone; ARR, aldosterone/renin ratio, * *P* < .05, ** *P* < .001

Changes in BP were −4.0 ± 1.6/−1.5 ± 1.1 mmHg in the first tertile of the ARR subgroup (ARR: ≤ 4.890), −8.8 ± 1.5/−3.8 ± 1.1 mmHg in the second tertile (ARR: 4.890–16.470), and −11.0 ± 1.3/−3.3 ± 0.9 mmHg in the third tertile (ARR: > 16.470). Changes in both the SBP and DBP among the high ARR group were greater than those among the low ARR group (*P* < .001 for change in SBP, *P* = .015 for change in DBP) (Figure 3A,B).

### 3.4 Comparison of changes in HBPM among the different subgroups according to baseline PRC, aldosterone concentration, and ARR

In the first tertile of the PRC subgroup, changes in BP were −8.9 ± 1.6/−7.4 ± 1.3 mmHg; in the second and third tertile, changes in BP were −5.7 ± 2.0/−3.5 ± 1.6 mmHg and −2.2 ± 1.9/−1.4 ± 1.5 mmHg, respectively. BP reduction was greater among the low PRC group than among the high PRC group (*P* = .002 for change in SBP, *P* = .016 for change in DBP).

The first tertile of the aldosterone subgroup recorded −6.9 ± 2.0/−3.3 ± 1.4 mmHg in BP changes; the second and third tertile subgroups recorded −6.9 ± 1.7/−4.4 ± 1.4 mmHg and −5.5 ± 2.0/−3.4 ± 1.8 mmHg in BP changes. There were no significant differences between the three subgroups based on baseline aldosterone concentration (*P* = .786 for change in SBP, *P* = .749 for change in DBP).

**FIGURE 4:**
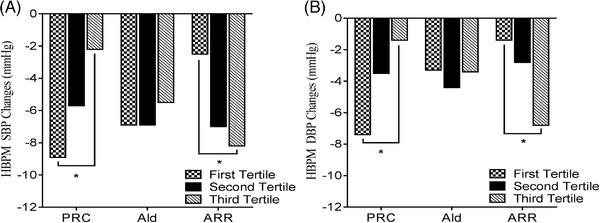
Comparison of HBPM changes in different subgroups according to baseline PRC, Ald, and ARR. Ald, aldosterone; ARR, aldosterone /renin ratio, * *P* < .05

Changes in BP among the ARR subgroup were −2.5 ± 1.9/−1.4 ± 1.5, −7.0 ± 2.0/−2.8 ± 1.6, and −8.2 ± 1.7/−6.8 ± 1.4 mmHg for the first, second, and third tertile, respectively. Changes in BP were greater among the high ARR group than among the low ARR group (*P* = .033 for change in SBP, *P* = .032 for change in DBP) (Figure 4A,B).

### 3.5 Safety evaluation of LS salt substitution by monitoring electrolyte concentrations and renal function

At baseline, we observed no significant differences in serum sodium and potassium concentrations between the two groups. At the endpoint of this study, the serum sodium level of the LS group was significantly lower than that of the NS group (*P* = .030), while the serum potassium level of the LS group was significantly higher than that of the NS group (*P* < .001). There were no significant differences in the BUN, Scr, and UA of the two groups at baseline and at the endpoint (*P* > .05) (Table 2). None of the participants suffered hyperkalemia or severe deterioration of renal function.

**TABLE 2:** Safety evaluation of LS salt substitution by monitoring electrolytes and renal function

	Baseline			Endpoint		
	NS	LS		NS	LS	
	(n = 160)	(n = 162)	*P*	(n = 160)	(n = 162)	*P*
Na (mmol/L)	140 (139, 141)	140 (139, 142)	.559	142 (140, 143)	141 (140, 142)	.030*
K (mmol/L)	4.04 (3.79, 4.27)	4.06 (3.85, 4.32)	.254	4.08 (3.88, 4.27)	4.23 (3.99, 4.45)	<.001**
BUN (mmol/L)	5.45 (4.76, 6.49)	5.72 (4.85, 6.46)	.392	4.99 (4.20, 6.03)	5.27 (4.53, 6.26)	.093
Scr (umol/L)	70.65 (57.60, 81.70)	64.75 (53.70, 79.03)	.075	71.45 (61.53, 84.40)	70.00 (59.73, 81.45)	.291
UA (umol/L)	332.45 (290.95, 388.58)	341.85 (288.50, 392.03)	.626	358.65 (294.98, 411.58)	354.10 (297.73, 405.40)	.607

NS, normal salt; LS, low‐sodium.

* *P* < .05.

** *P* < .001.

### 3.6 Evaluation of compliance by monitoring urine sodium and potassium levels

Baseline and endpoint measurements of urine sodium and potassium levels were used to evaluate the compliance of participants to using LS salt substitution. No differences were observed in the urine sodium and potassium levels of the two groups at baseline. At the endpoint, the urine sodium level of the LS group was significantly lower than that of the NS group [87.0 mmol/L (60.3, 123.5) vs 115.5 mmol/L (85.0, 143.0), *P* < .001], while the urine potassium level of the LS group was significantly higher than that of the NS group [36.4 mmol/L (23.7, 60.2) vs 31.4 mmol/L (20.6, 45.9), *P* = .012] (Table 3).

**TABLE 3:** Evaluation of compliance by monitoring urine sodium and potassium

	Baseline			Endpoint		
	NS	LS		NS	LS	
	(n = 160)	(n = 162)	*P*	(n = 160)	(n = 162)	*P*
Sodium (mmol/L)	106.5 (68.3, 143.8)	109.5 (72.5, 143.3)	.259	115.5 (85.0, 143.0)	87.0 (60.3, 123.5)	<.001**
Potassium (mmol/L)	64.7 (41.0, 84.2)	62.3 (44.7, 88.6)	.808	31.4 (20.6, 45.9)	36.4 (23.7, 60.2)	.012*

NS, normal salt; LS, low‐sodium.

* *P* < .05,.

** *P* < .001.

## 4 DISCUSSION

Our results demonstrate that only systolic OBPM was reduced by low‐sodium salt substitution. However, after 12 months of treatment, both systolic and diastolic HBPM were significantly reduced among middle‐ aged and elderly hypertensive patients. Diastolic OBPM was reduced, but not to a significant extent.

A previous clinical trial found that salt substitution significantly reduced office SBP by 8.2 mmHg and DBP by 3.4 mmHg.^18^ Another study conducted in northern China evaluated only the effect of salt substitution on HBPM and indicated that salt substitution decreased the systolic and diastolic HBPM of hypertensives and their family members.^19^ However, our study finds significant decrease in office SBP without an obvious reduction in DBP, which is consistent with another study that only found an effect on office SBP in hypertensives with diabetes.^20^ This may be because hypertension in the elderly is characterized by an elevated SBP, and the participants in our study were middle‐aged and elderly patients. Hence, the effect of LS salt substitution on DBP was relatively small. Secondly, there is a lack of conformity (42%) between OBPM and HBPM because of white‐coat and masked hypertension phenomena in OBPM,^21^ which also accounts for the underestimated BP lowing effect on OBPM^22^ and the inconsistent results of OBPM and HBPM.

A recent study that enrolled 2376 participants used the stepped‐wedge cluster randomized method to evaluate the effect of salt substitution and demonstrated that salt substitution decreased office SBP by 1.29 mmHg,^23^ which was a smaller value than that observed in our study. This may be because the LS salt substitution used in their study contained 75% NaCl, while ours is 43% NaCl. In addition, that study included participants with and without hypertension, while all the participants in our study are hypertensives. Researchers have reported that salt substitution treatment achieved a greater decrease in BP among hypertensives than among normotensives.^24^

This study presents the relationship of changes in BP with baseline PRC and ARR after LS salt substitution intervention. The changes in both systolic and diastolic OBPM and HBPM were significantly larger after LS salt substitution treatment for individuals with a low baseline PRC and high ARR than for those with a high baseline PRC and low ARR. A previous study showed that BP response was significantly greater in the patients with baseline low PRC than those with high baseline PRC after diuretic treatment,^25^ which also indicated that low PRC level can be used as a marker of high salt sensitivity. Salt sensitivity can be identified using the baseline PRC levels due to its relationship with renin^26^ owing to reduced glomerular filtration rate that influences renal sodium excretion and induces volume overload. The importance of salt sensitivity should be emphasized because of the potential harmful impact of salt consumption on individuals with high salt sensitivity. Salt sensitivity is associated with left‐ventricular hypertrophy and cardiovascular events, which has been deemed an independent risk factor for cardiovascular disease^27^ and mortality.^28^ Identifying salt sensitivity may help guide drug therapy^29^ and the implementation of salt restriction as a therapeutic method.

Individuals over 50 years old may have a higher salt intake which is due to age‐related taste bud deterioration.^5^ Thus, elderly patients were expected to have larger effects of LS salt substitution. Many young individuals prefer to eat at the restaurant or at their workplace, which may influence the effect of LS salt substitution on BP among this group. Therefore, we selected middle‐aged and elderly patients who ate at least two meals per day at home.

In our study, serum potassium levels increased among the two groups without chronic conditions such as hyperkalemia. Serum sodium levels also increased among both the NS group and the LS group. This may be attributable to conducting the last interview on a hot summer day, which caused patients to sweat—leading to volume loss.^30^ The BUN, Scr, and UA levels all changed without significance. No deterioration of renal function occurred. Thus, we can infer from this study that LS salt substitution is safe for long‐term intervention.

By monitoring urine sodium and potassium levels, at the endpoint of this study, we found that the urine sodium levels among the LS group were lower and the potassium levels were higher than the levels among the NS group, with a statistical significance. Therefore, we confirmed the compliance to LS salt substitution by participants in the intervention group.

## 5 LIMITATIONS

First, we evaluated the BP responses to LS salt substitution after intervention according to baseline PRC and ARR in middle‐aged and elderly participants but not in young participants. Second, we evaluated compliance to LS salt substitution by measuring fasting spot urine sodium and potassium levels, which may not provide the exact sodium and potassium intake.

## 6 CONCLUSIONS

In conclusions, LS salt substitution is effective in reducing systolic OBPM, systolic HBPM, and diastolic HBPM in middle‐aged and elderly hypertensive patients. LS salt substitution may offer a non‐pharmaceutical therapy for hypertensive patients. Baseline PRC may be a marker to predict BP response after salt restriction.

## CONFLICT OF INTEREST

There are no conflicts of interest.

## AUTHOR CONTRIBUTIONS

Yinong Jiang contributed to the conception and design of the study. Li Che contributed to writing the first draft of the manuscript. Wei Song contributed to the collection of data for the study. Ying Zhang and Yan Lu contributed to data analysis. Yunpeng Cheng contributed to interpretation for the study. All authors revised the manuscript and approved the final version.
